# Exploring Learned Surveillance Video Coding with Long-Term Reference and Adaptive Long–Short Modeling

**DOI:** 10.3390/s26082461

**Published:** 2026-04-16

**Authors:** Yuansheng Wu, Liangchao Hu, Xiaodan Song

**Affiliations:** 1 National Key Laboratory of Complex Aviation System Simulation, Southwest China Institute of Electronic Technology, Chengdu 610036, China; wuyuansheng@cetc.com.cn; 2 Guangzhou Institute of Technology, Xidian University, Guangzhou 510555, China; 22171214733@stu.xidian.edu.cn

**Keywords:** surveillance video coding, learned video coding, long-term reference, long–short correlation, adaptive motion modeling, adaptive context mining

## Abstract

Video coding plays a critical role for efficient transmission in surveillance camera sensors. Although long-term reference (LTR) has been fully studied in traditional hand-designed video coding approaches, its potential in learned video coding is still unexplored due to the highly unequal importance between long and short motion and the excessive motion overhead, especially for dense motion representation, e.g., optical flow. In this paper, we build an LTR baseline for learned surveillance video coding and propose an adaptive long–short modeling approach to address the above problem. Specifically, we first introduce LTR and propose a long–short context mining module to the authorized end-to-end video coding exploration model (EEM) from China’s AVS as a baseline. Since the quality of LTR significantly impacts its performance and importance, it is subsequently enhanced. Then, we propose a long–short motion adapter to address the unequal importance. Finally a historical motion guidance module is introduced to aid the motion decoding. Experimental results demonstrate that the proposed approach improves from a 1.86% BD-rate loss on EEM-4.1 to 13.89% BD-rate savings in YUV-PSNR compared with the anchor H.266/VVC under a low-delay P configuration. Although the current results are not comparable to the 44.01% gains of DCVC-FM, the proposed approach consumes less computational resources and we believe that integrating the proposed LTR method with stronger baselines will further boost the performance.

## 1. Introduction

The explosive growth of surveillance video sensors and their applications [1,2] has imposed a heavy burden on transmission bandwidth and compression algorithms [3,4,5,6]. Current mainstream video compression approaches, e.g., the H.26x series [7,8,9], Audio and Video coding Standard (AVS) series [10,11,12], and AOMedia Video (AV) series specifications  [13,14,15], primarily employ a hybrid prediction and transform coding scheme to eliminate redundancy. Surveillance video coding (SVC) mainly follows their frameworks and addresses the static background and dynamic foreground [16,17,18,19,20]. Particularly, the LTR tool has significantly improved the compression efficiency [21,22,23] via introducing a special frame that is kept for a much longer period, especially on surveillance videos. Despite the notable advances, the hand-designed frameworks have gradually reached a bottleneck over the past decades of development.

Recently, learned video compression (LVC) has achieved remarkable progress via end-to-end joint optimization. The framework mainly includes two schemes: residual coding (RC) [24,25,26,27,28] and conditional coding (CC) [29,30,31,32,33,34,35,36,37]. The latest work with CC has surpassed H.266/VVC with over 20% bitrate savings [33] under the low-delay P (LDP) configuration while achieving real-time encoding and decoding on GPUs [34] but lacking open source training code. China’s EEM (AVS group is now exploring a lightweight end-to-end learned video coding standard. EEM-x is the corresponding reference model. Its training and testing codes can be accessed on 11 April 2026 at https://openi.pcl.ac.cn/shengxihua/AVS-EEM); it aims for LVC standardization and is accessible via authorization, which accelerates its advancement and has significantly refined CC-based models to enhance efficiency, precision, and real-time performance. Although previous works [38,39,40,41] have studied surveillance videos with LVC, they mainly focus on static foreground and dynamic background modeling and their performance lags far behind existing learned approaches due to inferior baselines. In this paper, we employ the advanced EEM as our baseline and explore the potential of LTR in learned surveillance video coding (LSVC).

LTR features the introduction of an additional frame as a long-term reference, which is retained for much longer than short-term references (STRs). Compared with STRs, LTR is beneficial in scenarios with periodic motion, and static backgrounds and repeated content, which are common in surveillance videos. However, most existing LVC approaches only work with a single reference and can only explore long-term correlation at the decoder. M-LVC [42] employs historical frames to generate motion vector predictions (MVPs) for motion compensation. Ref. [43] proposes a long-term information exploitation module to dynamically update the long-term context with the reconstructed features from a previous reference. Similarly, ref. [44] introduces a long-term temporal context-gathering module that includes context clustering for classification and gathering to retrieve relevant context in an intra-cluster fashion. Ref. [45] interpolates a new reference given multiple frames without motion compression. Ref. [46] handles motion inconsistency and occlusion by decomposing the video into structure and detail components, recurrently accumulating the temporal information of each historical reference feature for long-term contexts and finally fusing them together. Despite improving performance, these decoder-side approaches may result in accumulated error propagation and ignore long-term correlation at the encoder, limiting their efficiency.

The methods with multiple references are still in their early stages and mainly focus on multiple STRs. Ref. [47] adopts a motion-free framework via interpolation and represents the current frame using a thumbnail and its residuals. Refs. [35,48,49] adopt an RC scheme and explore the long-term correlations like traditional methods. LHBDC [48] addresses the MVP for bi-directional motion compression. L-LBVC [35] focuses on accurate long–short motion estimation via recursive accumulation. Compared with multiple STRs, the use of mixed LTRs and STRs addresses the significant unequal importance between LTR and STR, including variations in quality and large-scale motions. Accurate selection between long-term and short-term correlations is critical. In traditional video coding, rate-distortion optimization (RDO) enables such adaptivity. However, RDO is notoriously non-differentiable. Existing LVC approaches typically lack an explicit RDO mechanism, instead treating the selection as a black box implemented with stacked networks, relying mainly on implicit learning to generate outputs given the inputs.

To address the above problems, we establish an LTR baseline for learned surveillance video coding and propose an adaptive long–short modeling approach, referred to as LSVC-LTR. Its core idea is that, instead of a hard selection, we introduce an attention mechanism as a soft selection to enable long–short adaptivity and refine the short-term context with the long-term information. Specifically, we employ EEM-4.1 as the base model and then introduce LTR with long–short context mining as the baseline. To leverage the advantage of LTR, we enhance its quality, which improves performance but exacerbates the unequal importance of different references. We further propose a long–short term motion adapter to adapt the long and short correlation. In addition, historical motion information is introduced during decoding to guide the reconstruction. Extensive experiments are established to verify the proposed method. In summary, the main contributions of this paper are as follows:

- We set up a new baseline for learned surveillance video coding with LTR and a long–short context mining module to exploit long-term correlations.
- A long–short motion adapter is proposed to address the unequal importance of LTR and STR. Furthermore, historical motion information is introduced to guide motion reconstruction.
- Extensive experimental results show the proposed LSVC-LTR surpasses H.266/VVC and achieves a 16.54% BD-rate saving compared with the baseline EEM-4.1 under the LDP configuration in terms of YUV-PSNR.

The remainder is organized as as follows: Section 2 presents the related work. The proposed LSVC-LTR is detailed in Section 3. Section 3.5 describes the training strategy. The experimental results are shown in Section 5. Section 7 concludes the paper.

## 2. Related Work

### 2.1. Learned General and Surveillance Video Compression

The manually designed traditional video coding approaches, including the H.26x series [7,8,9] developed by ITU-T and ISO/IEC, China’s AVSx [10,11,12], and AOM’s AV-series [13,14,15] standards, have gradually reached a bottleneck, relying on high computational complexity for high efficiency. Unlike this paradigm, learning-based video compression (LVC) employs neural networks (NNs) to enhance coding efficiency. Early works relied on the hybrid framework [50] and replaced partial modules. Later, end-to-end methods were introduced to benefit from joint optimization. Mainstream LVC can be classified into the RC [24,25,26,27,28] and CC [29,30,31,32,33,34,35] frameworks. RC adopts a framework with residual coding similar to traditional approaches, while CC models compression in a conditional coding fashion, in which correlation is modeled as context, resulting in lower entropy than RC. Currently, the most advanced LVC methods [34] with CC have surpassed the latest traditional coding standard, H.266/VVC, under the LDP configuration. Despite their advances, the lack of training code impedes their further development. Meanwhile, China’s End-to-end video coding Exploration Model (EEM) from AVS has started standardization efforts and refined CC-based models for efficiency, precision, and real-time performance. Particularly, its training source code is available once authorized.

The learned framework has also been extended to surveillance video coding but lags significantly behind that for general videos. Most approaches adopt the RC framework and mainly focus on strategies that separately encode the foreground and background. Wu et al. [38] separate the foreground/background via an adaptive Gaussian mixture model and adaptive updates or interpolation of shared backgrounds. Zhao et al. [39] propose an online mask-network separation method, compress foreground via RC frameworks, and use background networks to handle residuals and template updates. Zhao et al. [40] introduce a training-free method by dividing videos into GOPs, averaging the first GOP as the background, and extracting the foreground via subtraction. All of these methods focus on foreground–background modeling to boost reconstruction robustness in complex scenes. In this paper, we explore the potential of LTR and primarily focus on adaptive long–short correlation modeling.

### 2.2. Learned Long-Term Correlation Exploitation

Compression with LTR intrinsically belongs to long-term and short-term correlation modeling. We classify the existing approaches into two categories: single-reference and multiple-reference. Most LVC approaches fall into the former, which restricts long-term correlation exploration solely to the decoder. The core idea is to exploit already transmitted and reconstructed information to guide or enhance the overall compression. M-LVC [42] employs the historical frames to generate MVP and establish motion compensation (MC). Wang et al. [43] propose enhancing compression efficiency by fusing short-term information from neighboring frames with long-term features captured through a novel exploitation mechanism. By leveraging both contexts and utilizing multiple frames for advanced motion prediction and redundancy removal, the quality of contexts and motion compression is significantly improved. Ref. [44] introduces a long-term temporal context-gathering module including context clustering for classification and gathering to retrieve relevant contexts in an intra-cluster fashion. Ref. [45] employs interpolation to generate a new reference from the given frames without compressing motion into the bitstream. Sheng et al. [46] focus on motion inconsistency and occlusion, recurrently accumulating temporal information from each historical reference feature for long-term contexts and finally fusing the long and short contexts together. Despite the introduction of long-term correlations and their improved performance, the decoder-only setting limits further performance gains. In addition, their reliance on decoded information may lead to accumulated errors, particularly for long sequences.

The multi-reference approaches instead explore long-term correlations at both the encoder and decoder, compressing partial long-term information into the bitstream. Ref. [48] proposes the first hierarchical, bi-directional learned codec over fixed-size GOPs with an RC framework and bi-directional motion compensation. B-CANF [49] extends its previous augmented normalizing flow-based approaches and introduces B*-frames to unify the P and B frame compression. Ref. [47] exploits the hierarchical B-frame correlation in a scalable fashion without motion coding by using interpolation from the bi-directional references. Ref. [51] focuses on the joint optimization of P and B frame compression. LHBDC [48] estimates the bi-directional motion and encodes the residual motion after subtracting the MVP generated from the bi-directional references. L-LBVC [35] proposes an adaptive motion estimation module that estimates long-term and short-term optical flows via recursive accumulation instead of direct estimation. Both long and short motion vectors are compressed. Additionally, an adaptive motion prediction module reduces motion coding costs. Their promising results show the importance of long-term correlations. However, their references are typically restricted to two or three frames away from the current frame. The LTR is usually much further than that and thus exhibits significant unequal importance, especially when the quality of the LTR is enhanced for better reference. Traditional hand-designed methods rely on RDO to adapt between long and short correlations. However, RDO is non-differentiable. In this paper, we propose a long–short motion adapter and a context mining module to address this problem. In addition, historical motion information is introduced to guide motion decoding.

## 3. The Proposed Method

**Figure 1:**
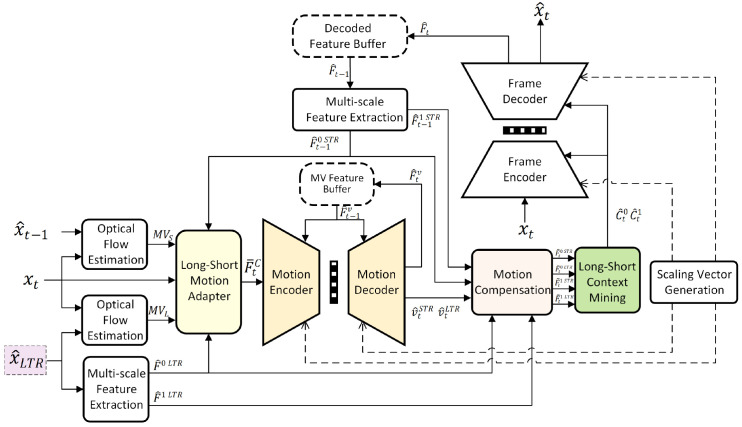
The overall architecture of the proposed LSVC-LTR for P frames. The intra-frame is compressed using existing learned image compression techniques. The colored modules are detailed due to the integration of the long-term reference. The remaining modules are either similar or identical to those in the baseline, i.e., EEM-4.1. from AVS of China.

This section introduces the proposed LSVC-LTR. The framework is built upon EEM-4.1 from AVS in China and is optimized for the LDP configuration by incorporating an LTR. The following subsections first describe the overall pipeline. Then the colored modules in Figure 1, including the Long-Term Reference, Long–Short Motion Adapter, Motion Encoder, Decoder with Historical Motion Vector Guidance, Compensation, and Long–Short Context Mining, are detailed in the context of LTR integration.

### 3.1. Overview

For a to-be-coded video, the frames are first divided into GOPs of equal length. The first frame within a GOP, referred to as an intra-frame (I-frame), is compressed using existing end-to-end image compression approaches. Particularly, instead of focusing on background and foreground modeling, we adopt the reconstructed intra-frame ${\hat{x}}_{LTR}$ as the LTR for universality and address the unequal importance between the LTR and STR. The quality of the LTR is critical to the overall performance; thus, we enhance the intra-frame quality to provide a better reference, which significantly boosts the overall performance. The reconstructed intermediate features of the intra-frame are stored in the decoded feature buffer until all frames within the GOP have been compressed. Subsequently, multi-scale features are first extracted from ${\hat{x}}_{LTR}$ to obtain ${\hat{F}}^{0\;LTR}$ and ${\hat{F}}^{1\;LTR}$, serving the subsequent motion modeling. The details are shown in Section 3.2. The remaining frames within a GOP are denoted as P frames under the the LDP configuration. Figure 1 shows the diagram of the proposed LSVC-LTR for P frame coding. It mainly includes the following parts:

Optical Flow Estimation. Given the current to-be-coded frame $x_{t}$, its previous reference ${\hat{x}}_{t-1}$, and the long-term reference ${\hat{x}}_{LTR}$, optical flow is employed to estimate the short-term motion vectors (MVs) $MV_{S}$ between $x_{t}$ and ${\hat{x}}_{t-1}$, and the long-term ones $MV_{L}$ between $x_{t}$ and ${\hat{x}}_{LTR}$.

Long–Short Motion Adapter. With the obtained motion vectors, i.e., $MV_{S}$ and $MV_{L}$, and the fine features from reconstructed images, i.e., ${\hat{F}}^{0\;LTR}$ and ${\hat{F}}_{t-1}^{0\;STR}$, the adapter adaptively weights the long-term and short-term motion features to address the varying contributions of different reference frames across different regions. Meanwhile, the original frame features are extracted from $x_{t}$ and concatenated with the weighted features, forming the compressed motion features ${\bar{F}}_{t}^{C}$. The details are shown in Section 3.4.

Motion Encoder, Decoder with Historical MV Guidance, and Compensation. The motion encoder and decoder take ${\bar{F}}_{t}^{C}$ as the input, conditioned on the motion features ${\hat{F}}_{t-1}^{v}$ from the previous frame, together with scaling coefficients $S_{mv}^{E}$ and $S_{mv}^{D}$ to compress and reconstruct the motion vectors ${\hat{v}}_{t}^{STR}$ and ${\hat{v}}_{t}^{LTR}$. The results, along with the long-term and short-term frame features, i.e., ${\hat{F}}^{0\;LTR}$, ${\hat{F}}^{1\;LTR}$, ${\hat{F}}_{t-1}^{0\;STR}$, and ${\hat{F}}_{t-1}^{1\;STR}$, are passed through the MC network to extract contextual information. Section 3.5 presents the details.

Long–Short Context Mining. Via the context mining network, the short-term features ${\tilde{F}}_{t}^{0\;STR}$ are refined by the long-term feature ${\tilde{F}}_{t}^{0\;LTR}$ to generate the final context ${\hat{C}}_{t}^{0}$. Similarly, the ${\tilde{F}}_{t}^{0\;LTR}$ is utilized to refine ${\tilde{F}}_{t}^{1\;STR}$ and generate small-scale contexts ${\hat{C}}_{t}^{1}$. The network structure is shown in Section 3.3.

Frame Encoder and Decoder. The frame encoder and decoder networks are responsible for compressing the current frame $x_{t}$ into a bitstream and reconstructing the current frame from the bitstream, respectively. The overall scheme adopts a conditional coding fashion and takes ${\hat{C}}_{t}^{0}$ and ${\hat{C}}_{t}^{1}$ from the MC network as conditions, which are hierarchically concatenated or fused with the intermediate features. After decoding, the reconstructed frame ${\hat{x}}_{t}$ is then obtained. Furthermore, the features ${\hat{F}}_{t}$ extracted before the last several layers of the frame decoder are stored in the decoded feature buffer and serve as reference features for the subsequent frames. Before serving as a reference, multi-scale features are extracted via a multi-scale feature extraction module. Similar to the motion codec, scaling coefficients are also generated from the scaling vector generation module to enable a variable bitrate. The detailed network structure can be found in the source code of EEM-4.1 (https://openi.pcl.ac.cn/shengxihua/AVS-EEM (accessed on 11 April 2026), which requires authorization).

Scaling Vector Generation (SVG). The SVG defines four sets of vectors, with each set corresponding to a different quality level, i.e., different rates. Each set of vectors contains six learnable quantization parameters: the motion quantization vector $S_{mv}^{E}$ and $S_{mv}^{D}$ for the motion encoder and decoder, respectively, and the frame quantization vectors $S_{frame}^{E}$ and $S_{frame}^{D}$ for the frame encoder and decoder. Once learned, the coefficients within SVG are constant.

### 3.2. Long-Term Reference

**Figure 2:**
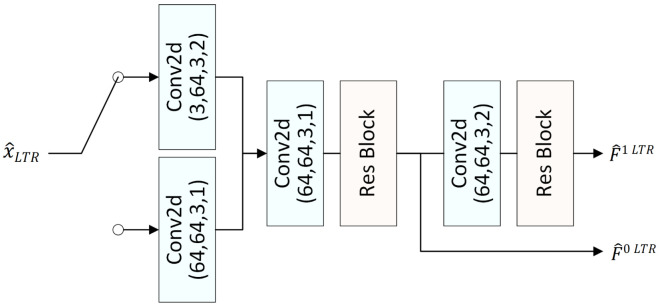
The multi-scale feature extraction network architecture. It is used to extract features from the long-term reference ${\hat{x}}_{LTR}$ (upper branch) and the feature ${\hat{F}}_{t-1}$ from the decoded feature buffer (lower branch).

We adopt the intra-frame of each GOP as the LTR and extend EEM-4.1 via replicating the pipeline for the short P reference frame. Considering the significance of reference quality in SVC, and the fact that the LTR is frequently utilized, we manually increase the bitrate of the intra-frame to benefit the subsequent P frame compression. The learned image compression approach in EEM-4.1 is employed to compress the intra-frame. The reconstructed intra-frame then serves as the LTR ${\hat{x}}_{LTR}$ for the remaining frames within the GOP, facilitating optical flow estimation for motion modeling and guiding the subsequent motion compression and compensation. For better guidance, the small-scale features ${\hat{F}}^{0\;LTR}$ and large-scale features ${\hat{F}}^{1\;LTR}$ are extracted via the multi-scale feature network shown in Figure 2. The implementation details for intra-frame compression can be found in the final paragraph of Section 5.1.

### 3.3. Long–Short Context Mining

EEM-4.1 is a conditional framework, in which the context is vital for the overall performance. Relying solely on the pipeline of STR makes it challenging to generate effective contexts. Thus, we propose the long–short context miming (LSCM) module, which aims to fuse predictive features from both long-term and short-term reference frames at the same scale to generate more accurate predictive features as contextual information for subsequent conditional coding. The fusion strategy primarily relies on predictive features from the STR and enhances them via a residual architecture incorporating the LTR. The process of predictive feature fusion can be described by

(1) $${\hat{C}}_{t}^{i}={\tilde{F}}_{t}^{i\;STR}+f_{fusion}\left({\tilde{F}}_{t}^{i\;STR},{\tilde{F}}_{t}^{i\;LTR}\right),$$

where *i* is either 0 or 1, with 0 and 1 denoting the small and large scales, respectively. $f_{fusion}\left(\cdot \right)$ represents the feature fusion network, and ${\hat{C}}_{t}^{i}$ denotes the finally generated contextual information.

**Figure 3:**
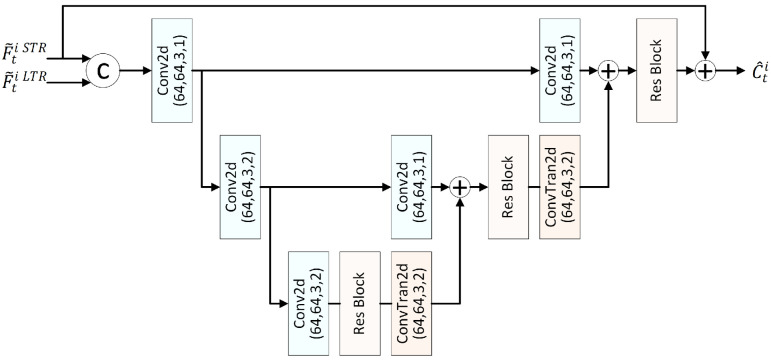
The network structure of the long–short context mining (LSCM) module, in which i=0,1.

LSCM aims to fuse the long-term and short-term contexts. Despite many choices, e.g., the deformable convolutions, a U-Net structure [52] is adopted to restrict the computational complexity. Figure 3 shows the network architecture of the LSCM module. A symmetric structure consisting of multi-level downsampling and upsampling is employed to capture multi-scale features. Meanwhile, features from the downsampling path are passed through a convolutional layer and then added to features in the upsampling path. During the upsampling process, a residual block, termed Res Block, is applied at each resolution level after the feature addition. To this end, we have built the LTR baseline.

### 3.4. Long–Short Motion Adapter

Compared to multiple STRs, the mixture of LTR and STR exhibits obviously unequal importance: the contribution of different reference frames varies significantly across different regions. For example, the LTR frame ${\hat{x}}_{LTR}$ has higher reference quality in regions with low temporal variation, e.g., backgrounds, while the STR frame ${\hat{x}}_{t-1}$ is strongly correlated with foreground moving objects. Traditional block-level rate distortion optimization (RDO) can partially address this issue. However, in LVC frameworks, a homogeneous processing strategy is often adopted, neglecting such differences due to the lack of explicit syntax element support and the non-differentiable property of RDO. In addition, enhancing LTR quality will further magnify this unequal importance. To address this issue, we introduce the long–short motion adapter (LSMA), aiming to mimic the adaptive selection and generate rich motion features ${\bar{F}}_{t}^{C}$ to facilitate the subsequent motion compression.

**Figure 4:**
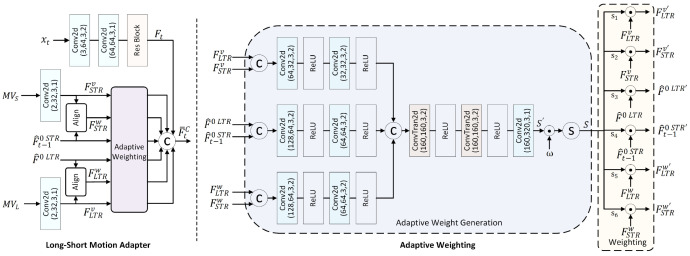
The architecture of the long–short motion adapter (LSMA) module. The left shows the overall scheme of LSMA, and the right shows the diagram of the adaptive weighting module. Its core idea lies in adaptively generating coefficients to weight the long-term and short-term motion features.

Figure 4 shows the detailed structure of the LSMA. Considering that the motion information is correlated with the target bitrate, e.g., motion information at lower bitrates is usually coarser to achieve better rate-distortion performance, we incorporate not only the long-term and short-term motion vectors $MV_{L}$ and $MV_{S}$, but also the current frame $x_{t}$ and reference frame features, i.e., ${\hat{F}}^{0\;LTR}$ and ${\hat{F}}_{t-1}^{0\;STR}$, as inputs. First, the current frame $x_{t}$, long-term motion $MV_{L}$, and short-term motion $MV_{S}$ are transformed to the feature space, obtaining $F_{t}$, $F_{LTR}^{v}$, and $F_{STR}^{v}$, respectively. Meanwhile, the alignment network warps the LTR frame feature ${\hat{F}}^{0\;LTR}$ and STR frame feature ${\hat{F}}_{t-1}^{0\;STR}$, resulting in the aligned features $F_{LTR}^{w}$ and $F_{STR}^{w}$. Next, an adaptive weighting network assigns weights to the motion features $F_{LTR}^{v}$ and $F_{STR}^{v}$, reference frame features ${\hat{F}}^{0\;LTR}$ and ${\hat{F}}_{t-1}^{0\;STR}$, and aligned features $F_{LTR}^{w}$ and $F_{STR}^{w}$. The resulting weighted features and the current frame feature $F_{t}$ are concatenated along the channel dimension to generate the joint feature ${\bar{F}}_{t}^{C}$. Consequently, the LSMA can be formulated as

(2) $${\bar{F}}_{t}^{C}=f_{LSMA}\left(x_{t},MV_{S},MV_{L},{\hat{F}}_{t-1}^{0\;STR},{\hat{F}}^{0\;LTR}\right).$$

${\bar{F}}_{t}^{C}$ denotes the concatenation of $F_{t}$, $F_{STR}^{v^{\prime }}$, $F_{LTR}^{v^{\prime }}$, ${\hat{F}}_{t-1}^{0\;STR^{\prime }}$, ${\hat{F}}^{0\;LTR^{\prime }}$, $F_{STR}^{w^{\prime }}$, and $F_{LTR}^{w^{\prime }}$, which represent the features extracted from the current frame, weighted short-term MV features, long-term MV features, short-term frame features, long-term frame features, warped short-term frame features, and warped long-term features, respectively. The core of the LSMA is the adaptive weighting sub-module shown in the right panel of Figure 4. It mainly includes:

(1) Adaptive Weight Generation (AWG). The inputs to AWG comprise three types of key features from long-term and short-term reference frames: motion features $F_{STR}^{v}$ and $F_{LTR^{\prime }}^{v}$, reference frame features ${\hat{F}}_{t-1}^{0\;STR}$ and ${\hat{F}}^{0\;LTR}$, and aligned features $F_{STR}^{w}$ and $F_{LTR}^{w}$. We adopt a bottleneck structure consisting of downsampling followed by upsampling to enlarge the receptive field. To reduce computational complexity, the network employs a grouped processing strategy, where the inputs are divided into three groups based on their types. Each group undergoes independent downsampling through two convolutional layers, followed by channel concatenation at the bottleneck layer, and then is restored to the original resolution through transposed convolutions. Subsequently, a spatial element-wise weight $S^{\prime }$ is generated, which is denoted as

(3) $$S^{\prime }=f_{AWG}\left(F_{LTR}^{v},\;F_{STR}^{v},\;{\hat{F}}^{0\;LTR},\;{\hat{F}}_{t-1}^{0\;STR},\;F_{LTR}^{w},\;F_{STR}^{w}\right).$$

$f_{AWG}$ denotes the feature extraction mentioned above.

Furthermore, to adapt to the global characteristics at different bitrates, a learnable parameter vector $\omega \in R^{C}$ is introduced to provide control across the channel dimension. This forces the network to preferentially allocate bitrates to high-weight regions, such as the background motion field of the LTR or the motion edges of the STR, while suppressing redundant information in low-contribution regions. The value of ω is related to the rate-distortion trade-off parameter λ, and its channel dimension *C* matches that of $S^{\prime }$, i.e., 6. After that, $S^{\prime }$ is multiplied by ω and then passed through the Sigmoid function σ(·) to obtain the final weight map *S* as

(4) $$S=\sigma \left(\begin{array}{c} S^{\prime }⊙\omega \end{array}\right).$$

(2) Weighting. With the spatial and channel adaptive weights *S*, the six input features are re-weighted as

(5) $$F_{i}^{\prime }=F_{i}⊙s_{i},$$

in which $F_{i}\in \left\{F_{LTR}^{v},F_{STR}^{v},{\hat{F}}^{0\;LTR},{\hat{F}}_{t-1}^{0\;STR},F_{LTR}^{w},F_{STR}^{w}\right\}$, and i=1,2,…,6. $F_{i}^{\prime }$ is the corresponding weighted feature of $F_{i}$. $s_{i}$ is obtained via

(6) $$s_{1},s_{2},\ldots ,s_{6}=Split\left(S\right),$$

where Split(·) represents channel splitting.

### 3.5. Motion Encoder, Decoder with Historical MV Guidance, and Compensation

**Figure 5:**
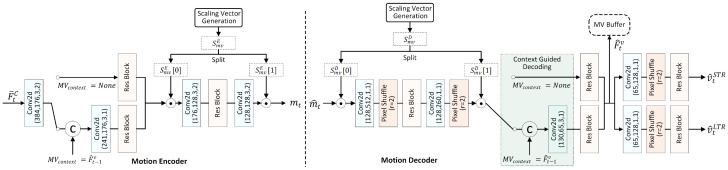
Left: the motion encoder network. Right: the motion decoder network. It should be noted that the distribution estimation network for entropy and quantization is not shown. They are similar to that in most learned image compression methods. The switch is controlled by the MV context. When t=1 and the reference is an intra-frame, the context does not exist.

Motion Encoder and Decoder with Historical MV Guidance. The motion encoder compresses the implicit representation ${\bar{F}}_{t}^{C}$ obtained from LSMA, while the motion decoder reconstructs the long-term and short-term motion vectors from the bitstream. Instead of using them merely for guidance, we explicitly encode both long-term and short-term motion information into the bitstream to mitigate error propagation. Figure 5 shows the network structure of the motion encoder (left) and motion decoder (right). The original structure from EEM-4.1 is largely retained, with three key modifications. At the encoder, the dimension of the first convolutional layer is increased to accommodate the concatenated LTR motion, frame features, and aligned ones. At the decoder, a dual-branch architecture is adopted to simultaneously generate the reconstructed LTR MV ${\hat{v}}_{t}^{LTR}$ and STR MV ${\hat{v}}_{t}^{STR}$. Notably, we observe that the historical motion feature ${\hat{F}}_{t-1}^{v}$ is only exploited at the encoder while being neglected at the decoder. Thus, the green-colored module in the motion decoder of Figure 5 is introduced to enhance the reconstructed MVs. The feature ${\hat{F}}_{t-1}^{v}$ is concatenated with the scaled features, followed by a feature extraction. Considering the lack of context when the reference is an intra-frame, we introduce a switch. The experimental results demonstrate its effectiveness.

**Figure 6:**
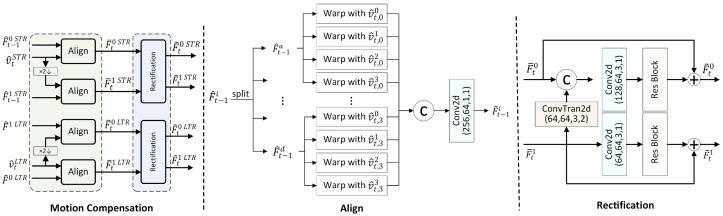
The motion compensation network. (**Left**): the overall diagram for long and short MC. (**Middle**): the details of the align sub-module. (**Right**): the rectification sub-module.

Motion Compensation. Figure 6 shows the network architecture of the motion compensation (MC), which aims to generate contexts for the frame codec. To exploit the long-term correlations, we replicate the STR pipeline for the LTR path within the MC module. Specifically, using the decoded MVs ${\hat{v}}_{t}^{LTR}$ and ${\hat{v}}_{t}^{STR}$, the large-scale and small-scale frame features are warped for alignment. Subsequently, the warped features are rectified between the small and large scales via the rectification network. Finally, we obtain the compensated features, i.e., ${\tilde{F}}_{t}^{0\;STR}$, ${\tilde{F}}_{t}^{1\;STR}$, ${\tilde{F}}_{t}^{0\;LTR}$, and ${\tilde{F}}_{t}^{1\;LTR}$.

## 4. Inference and Training Details

The core of this paper lies in the compression of P frames with an LTR. Algorithm 1 summarizes the overall encoding and decoding process for inference. During training, only the network shown in Figure 1 is trained, while the intra-frame codec adopts the pre-trained model from EEM-4.1. The training process is divided into two major stages: pre-training and fine-tuning. The loss functions for different stages vary depending on the specific network components. The AdamW optimizer is employed across all training stages, with $\beta_{1}=0.9$ and $\beta_{2}=0.999$. In the pre-training stage, the PWC-Net [53] is used for optical flow estimation, whereas in the fine-tuning training stage, the lightweight FastFlowNet [54] is adopted. Notably, the optical flow estimation networks in both stages are frozen and do not participate in the training process. The Vimeo-90k [55] dataset is utilized for pre-training, whereas the BVI-DVC [56] dataset is employed during fine-tuning. The training strategy for the proposed LSVC-LTR closely follows that in EEM-4.1, except for fine-tuning. More details can be found in the source code of EEM-4.1.

The fine-tuning stage adopts YUV-MSE as the main distortion metric, replacing the RGB-MSE used in the pre-training stage. It primarily includes three sub-stages: (1) The GOP size is set to 7 with a learning rate (lr) of 5 $\times \;10^{-6}$; (2) the GOP size is increased to 9; and (3) the lr is decreased to 1 $\times \;10^{-7}$. Due to the limited availability of surveillance video datasets, we do not use dedicated surveillance data for training. Instead, a repeated compression training strategy is employed to simulate surveillance scenarios using general video datasets. The core idea is that surveillance content remains static for extended periods in the extreme case. Specifically, we repeat the first frame by *k* times and then append the subsequent frames sequentially. This modified sequence then serves as the training data. For robustness, we randomly sample k∈[0,40] for each sequence. It should be noted that this strategy is only applied during sub-stages (2) and (3) of the fine-tuning stage.
**Algorithm 1** The proposed learned surveillance video coding with long-term reference (LSVC-LTR) inference algorithm for a video with a $GOP_{size}$ length.  1:**Input:** A video sequence $\left\{x_{t}\right\}$, t∈[0,$GOP_{size}-1$]; A pre-trained inter model $M_{inter}$; A pre-trained intra model $M_{intra}$  2:**Output:** Compressed binary bitstream Bits, Reconstructed sequence $\left\{{\hat{x}}_{t}\right\}$  3:/*************Initialization*************/  4:Initialize Bits←{}, ${\hat{F}}_{0}^{v}\leftarrow None$  5:$\left\{S_{mv}^{E},S_{mv}^{D},S_{frame}^{E},S_{frame}^{D}\right\}\leftarrow ScalingVectorGeneration\left(None\right)$  6:/*************Intra Frame Encoding and Decoding*************/  7:$Bits_{intra}\leftarrow IntraEncoder_{M_{intra}}\left(x_{0}\right)$  8:$Bits\leftarrow Append(Bits,Bits_{intra})$  9:${\hat{x}}_{0}\leftarrow IntraDecoder_{M_{intra}}\left(Bits_{intra}\right)$10:/*************Inter Frame Encoding and Decoding*************/11:${\hat{x}}_{LTR}\leftarrow {\hat{x}}_{0}$12:${\hat{F}}_{0}\leftarrow {\hat{x}}_{0}$13:$\left\{{\hat{F}}^{0\;STR},{\hat{F}}^{1\;STR}\right\}\leftarrow MultiScaleFeature\left({\hat{x}}_{LTR}\right)$14:**for** $t=1,\ldots ,GOP_{size}-1$ **do**15:   $\left\{{\hat{F}}_{t-1}^{0\;STR},{\hat{F}}_{t-1}^{1\;STR}\right\}\leftarrow MultiScaleFeature\left({\hat{F}}_{t-1}\right)$16:   /******************Motion******************/17:   $MV_{S}\leftarrow OpticalFlow\left(x_{t},{\hat{x}}_{t-1}\right)$, $MV_{L}\leftarrow OpticalFlow\left(x_{t},{\hat{x}}_{LTR}\right)$18:   $\left\{{\hat{F}}^{0\;LTR},{\hat{F}}^{1\;LTR}\right\}\leftarrow MultiScaleFeature\left({\hat{x}}_{LTR}\right)$19:   ${\bar{F}}_{t}^{C}\leftarrow LongShortMotionAdapter\left(x_{t},MV_{S},MV_{L},{\hat{F}}_{t-1}^{0\;STR},{\hat{F}}^{0\;LTR}\right)$20:   $Bits_{motion}\leftarrow MotionEncoder\left({\bar{F}}_{t}^{C},{\hat{F}}_{t-1}^{v},S_{mv}^{E}\right)$21:   $Bits\leftarrow Append(Bits,Bits_{motion})$22:   $\left\{{\hat{v}}_{t}^{LTR},{\hat{v}}_{t}^{STR},{\hat{F}}_{t}^{v}\right\}\leftarrow MotionDecoder\left(Bits_{motion},{\hat{F}}_{t-1}^{v},S_{mv}^{D}\right)$23:   $\left\{{\tilde{F}}_{t}^{0\;STR},{\tilde{F}}_{t}^{1\;STR},{\tilde{F}}_{t}^{0\;LTR},{\tilde{F}}_{t}^{1\;LTR}\right\}\leftarrow MotionCompensation({\hat{v}}_{t}^{LTR},{\hat{v}}_{t}^{STR},$24:   $\;\;\;\;\;\;\;\;\;\;\;\;\;\;\;\;\;\;\;\;\;\;\;\;\;\;\;\;\;\;\;\;\;\;\;\;\;\;\;\;\;\;{\hat{F}}_{t-1}^{0\;STR},{\hat{F}}_{t-1}^{1\;STR},{\hat{F}}^{0\;LTR},{\hat{F}}^{1\;LTR})$25:   /************Context Mining************/26:   $\left\{{\hat{C}}_{t}^{0},{\hat{C}}_{t}^{1}\right\}\leftarrow LongShortContextMining\left({\tilde{F}}_{t}^{0\;STR},{\tilde{F}}_{t}^{1\;STR},{\tilde{F}}_{t}^{0\;LTR},{\tilde{F}}_{t}^{1\;LTR}\right)$27:   /***Frame Encoding and Decoding***/28:   $Bits_{frame}\leftarrow FrameEncoder\left(x_{t},{\hat{C}}_{t}^{0},{\hat{C}}_{t}^{1},S_{frame}^{E}\right)$29:   $Bits\leftarrow Append(Bits,Bits_{frame})$30:   $\left\{{\hat{x}}_{t},{\hat{F}}_{t}\right\}\leftarrow FrameDecoder\left(Bits_{frame},{\hat{C}}_{t}^{0},{\hat{C}}_{t}^{1},S_{frame}^{D}\right)$31:**end for**32:**return** Bits, $\left\{{\hat{x}}_{t}\right\}$

## 5. Experimental Results

### 5.1. Experimental Setting

**Figure 7:**
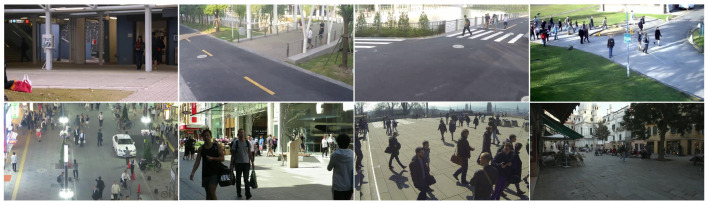
The test sequences include 01 from the Avenue Dataset test set; 05 and 07 from the ShanghaiTech Campus dataset test set; PETS09-S2L2-raw from MOT15; MOT17-01-SDP, MOT17-03-SDP, and MOT17-08-SDP from MOT17; and cam3 from the WILDTRACK Dataset.

To evaluate the performance of the proposed LSVC-LTR, we select test sequences from the Avenue Dataset [57], the ShanghaiTech Campus dataset [58,59], MOT15, MOT17, and the WILDTRACK Dataset [60], which are shown in Figure 7. These sequences have 1920 × 1080 resolution and cover both typical surveillance scenarios and complex public areas, e.g., campuses, urban streets, and squares. By containing diverse behaviors and environmental characteristics, they enable a comprehensive evaluation. The PETS09-S2L2-raw and MOT17-01-SDP sequences are tested up to their maximum number of frames, i.e., 436 and 450 frames, respectively. The remaining sequences are tested for 600 frames.

For traditional video compression methods, we compare our approach with the official reference software HM-18.0 for H.265/HEVC and VTM-22.0 for H.266/VVC. The QP values are set to 29, 32, 35, and 40. For end-to-end LVC video coding approaches, we compare the proposed method with state-of-the-art (SOTA) methods, namely DCVC-DC [32], DCVC-FM [33], and our baseline EEM-4.1 from AVS. The test GOP size is set to 32 under the LDP configuration. BD-rate is used to evaluate the coding performance, where a negative value indicates a bitrate reduction at equivalent quality. BD-rate is calculated for each component in the YUV color space, and a weighted “YUV” BD-rate is also provided using a 6:1:1 ratio. VTM-22.0 is employed as the anchor for BD-rate calculations. During training, MSE is used as the image distortion loss, while PSNR is primarily used for performance evaluation, with MS-SSIM serving as a supplementary metric. Since our codec is designed as a general-purpose compression framework rather than one specifically optimized for video coding for machines, the task-driven metrics, e.g., object detection accuracy, are not reported. For convenience, the aforementioned eight test sequences are denoted as Seq1, Seq2, …, Seq8 in the following tables.

For the proposed LSVC-LTR, P frames are compressed by the proposed method in Figure 1. Intra-frames are compressed by the pre-trained models provided by EEM-4.1, but with an adjusted bitrate, considering their frequent use as references. However, while higher quality improves reference quality, it also leads to excessively high bitrates. Therefore, we only shift to the next higher rate-point to limit the bitrate overhead. In our implementation, the pre-trained model that has a higher rate and is next to the default model is selected to compress the intra-frame. We observe that the PSNR of the LTR is typically 0.5~0.8 dB higher than that of the STRs. All experiments are conducted on a server equipped with a 14th Gen Intel Core i7-14700F CPU and dual NVIDIA GeForce RTX 4090 GPUs, each with 24 GB memory.

### 5.2. Comparison with State-of-the-Art

Table 1 shows the BD-rate comparison among the proposed method, SOTA methods, and the baseline EEM-4.1 in terms of PSNR, with VTM-22.0 serving as the anchor. The proposed LSVC-LTR demonstrates average bitrate savings of 13.89% for weighted YUV, and 0.16%, 53.50%, and 56.66% for the Y, U, and V components, respectively, over the latest traditional H.266/VVC, i.e., VTM-22.0. Significant bitrate savings are achieved compared to HM-18.0, improving from a 32.34% increase to a 13.89% reduction. Compared with our baseline EEM-4.1, LSVC-LTR converts the 1.86% loss to significant gains. Specifically, the BD-rate is improved from 12.41%, −28.78%, −30.79%, and 1.86% to −0.16%, −53.50%, −56.66%, and −13.89% for the Y, U, V, and weighted YUV components, respectively. Table 2 shows the performance comparison on MS-SSIM. The LVC methods consistently outperform the traditional ones. The proposed method achieves BD-rates of −11.15%, −34.43%, −36.58%, and −17.24% relative to VTM-22.0 for the Y, U, V, and YUV components. Compared with EEM-4.1, the BD-rate improves from a 3.65% loss to a 17.24% gain, achieving approximately 20.89% in additional bitrate savings.

**Figure 8:**
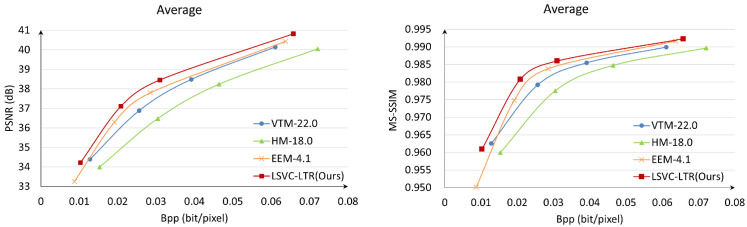
The averaged RD curve for PSNR (**Left**) and MS-SSIM (**Right**).

**Figure 9:**
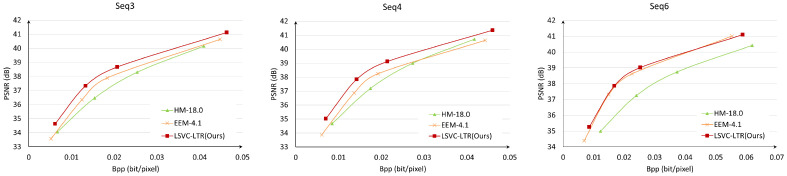
The RD curves for different sequences, i.e., Seq3 (**Left**), Seq4 (**Middle**), and Seq6 (**Right**).

Figure 8 shows the averaged RD curves over the test data in terms of PSNR and MS-SSIM. It can be observed that the proposed LSVC-LTR outperforms the other three methods. Compared with EEM-4.1, LSVC-LTR consistently performs better across all four tested rate points. In Figure 9, we select three sequences to show the detailed RD curves across different rates. The results show that the performance gain varies across sequences, where Seq3 and Seq4 achieve large gains over EEM-4.1. However, for Seq6, the improvement is marginal, and some loss is observed at the highest bitrate. This may be attributed to the foreground motion, as shown in Figure 7, which covers a large area of the frame.

Compared with DCVC-DC and DCVC-FM, the proposed LSVC-LTR still lags behind. Particularly, it can be observed that DCVC-FM achieves up to 80% bitrate savings on U and V components over VVC, indicating the superiority of LVC approaches. One reason may be that, in traditional hand-designed approaches, e.g., H.266/VVC, less attention is paid to the optimization of chroma components compared to the Y component, which is prioritized due to its higher sensitivity to human vision. This also suggests that there is still significant room for improvement regarding the chroma components. However, the computational complexity and the parameters of the proposed method are significantly lower than those of the aforementioned methods. As shown in Table 2, the Multiply–Accumulate Operations (MACs) of the proposed LSVC-LTR are 1457G, and the number of parameters is 13.81M, accounting for only 55.1% and 74.9% of DCVC-DC, and 65.5% and 81.1% of DCVC-FM, respectively. Additionally, due to the challenges of reproduction, most existing methods published after DCVC-DC and DCVC-FM also exhibit a certain gap when compared with these two approaches. Compared with the baseline EEM-4.1, the MACs are doubled since two references, i.e., the STR and LTR, are adopted, requiring repeated optical flow estimation, compression, compensation, and context mining. The complexity may be further refined via exploiting the correlation among long-term motions across different frames.

### 5.3. Visual Quality

**Figure 10:**
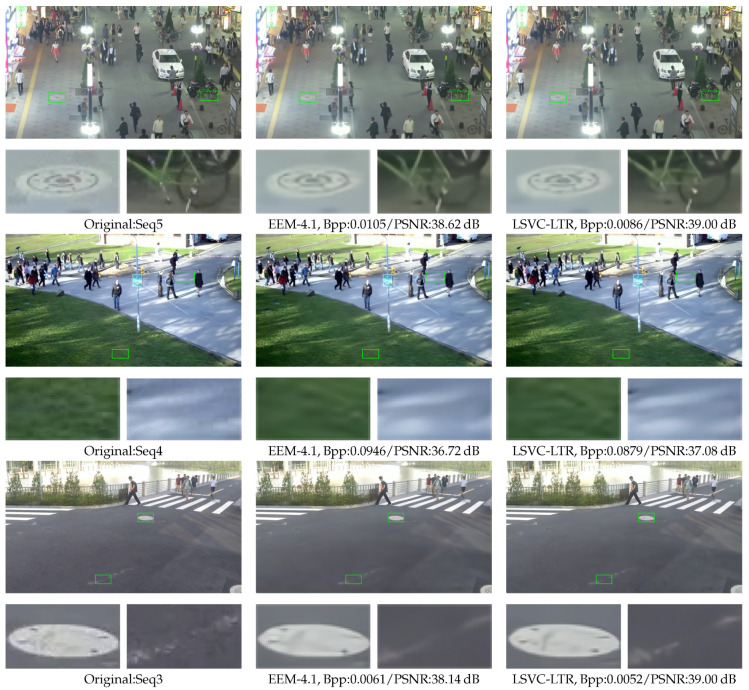
The visual quality comparison between EEM-4.1 and LSVC-LTR on Seq5, Seq4, and Seq3. The marked regions are zoomed in and shown below the corresponding frame.

To evaluate the subjective quality of the proposed method, three test sequences, i.e., Seq3, Seq4, and Seq5, are presented in Figure 10. It can be observed that the reconstructed details and textures, e.g., the dots on the street manhole cover, the tire, the grass, and the line on the road, are much clearer compared to those of EEM-4.1. For Seq5, the Bpp is reduced from 0.105 to 0.0086 with about a 18.10% bitrate reduction, while the reconstructed PSNR improves by 0.28 dB compared with EEM-4.1. Specifically, for Seq3, the rate saving reaches 14.75% and the reconstruction quality is significantly enhanced by 0.86 dB. Similarly, the bitrate reduction for Seq4 is 7.08% with a 0.36 dB improvement in PSNR, largely due to better reconstruction of the grass regions. These results demonstrate that the proposed LSVC-LTR effectively enhances the quality of reconstructed images while simultaneously reducing the bitrate.

### 5.4. Performance Analysis

In this section, we conduct a detailed performance analysis to demonstrate the validity of the proposed LSVC-LTR from the following aspects:

Ablation Studies. To verify the effectiveness of each component of the LSVC-LTR, ablation studies are conducted on Seq5 and Seq7. With EEM-4.1 adopted as the anchor, each component is gradually integrated. The results are shown in Table 3. By comparing row 1 and row 2, it can be observed that the introduction of LTR, combined with the long–short context mining (LSCM) module, achieves a 7.97% bitrate saving. By enhancing LTR, the bitrate reduction further reaches −13.84%, representing an additional 5.87% saving by comparing row 2 and row 3. Furthermore, incorporating historical MV guidance into the motion decoder enables additional 0.67% rate savings, comparing row 3 with row 4. Finally, the inclusion of the critical long–short motion adapter (LSMA) boosts the overall performance from −14.51% in row 4 to −16.54% in row 5, yielding an improvement of 2.03%. The results clearly demonstrate the individual effectiveness of each component in LSVC-LTR.

Adaptive Weights Analysis of LSMA. The weight map generated by the LSMA incorporates spatial fine-grained control and channel-level control ω, which aims to adapt to the global characteristics at different bitrate points. To demonstrate how the weighting network operates, this subsection analyzes ω and the final weights *S*.

**Figure 11:**
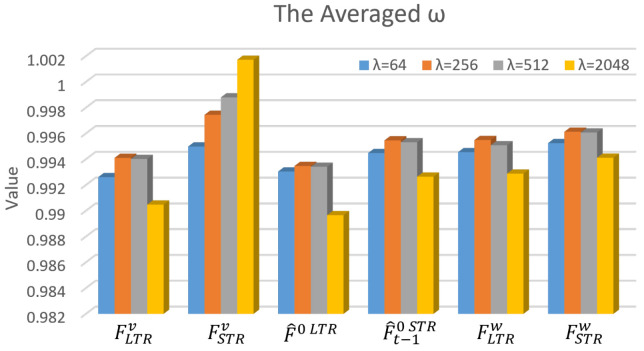
The averaged weight ω across channels for different features, i.e., the LTR motion feature $F_{LTR}^{v}$, the STR motion feature $F_{STR}^{v}$, the LTR frame feature ${\hat{F}}^{0\;LTR}$, the STR frame feature ${\hat{F}}^{0\;STR}$, the warped LTR feature $F_{LTR}^{w}$, and the warped STR feature $F_{STR}^{w}$ in Figure 4. λ controls the overall rate for a video, and a larger value indicates a higher bitrate.

In ω, each value corresponds to a channel weight for the six weighted features, i.e., $F_{LTR}^{v}$, $F_{STR}^{v}$, ${\hat{F}}^{0\;LTR}$, ${\hat{F}}_{t-1}^{0\;STR}$, $F_{LTR}^{w}$, and $F_{STR}^{w}$. The channel dimension for each feature is set to 32 for $F_{LTR}^{v}$ and $F_{STR}^{v}$, 64 for ${\hat{F}}^{0\;LTR}$ and ${\hat{F}}_{t-1}^{0\;STR}$, and 64 channels for $F_{LTR}^{w}$ and $F_{STR}^{w}$, totaling 320 channels that correspond to the 320 weight values in ω. We average the weights across channels for the same type of features, e.g., averaging the 32 weights for $F_{LTR}^{v}$. Figure 11 shows the averaged results for the six types of features, in which λ controls the bitrate, and a larger value indicates a higher rate. It can be observed that weights for the STR motion feature $F_{STR}^{v}$ generally increase with the bitrate and are consistently larger than those of LTRs. This indicates the dominant role of the STR motion, while the LTR provides additional auxiliary information. Similar trends can be observed in the reference frame features ${\hat{F}}^{0\;LTR}$ and ${\hat{F}}_{t-1}^{0\;STR}$ and the warped frame features $F_{LTR}^{w}$ and $F_{STR}^{w}$. Furthermore, it can be observed that the weights for warped LTR frame features exceed those of the unaligned ones.

**Figure 12:**
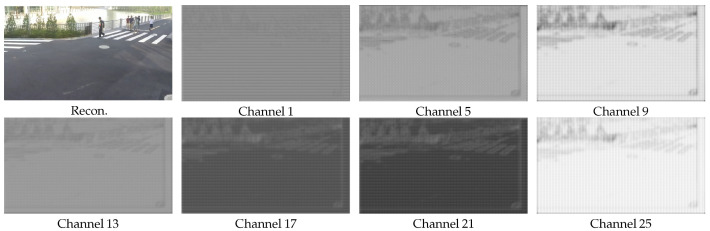
The overall weight $s_{1}$ visualization for the LTR motion feature $F_{LTR}^{v}$, in which λ=2048.

*S* further provides spatial-level weighting based on the channel weights ω, comprising six elements—$s_{1}$, $s_{2}$, $s_{3}$, $s_{4}$, $s_{5}$, and $s_{6}$—which correspond to the weights of $F_{LTR}^{v}$, $F_{STR}^{v}$, ${\hat{F}}^{0\;LTR}$, ${\hat{F}}_{t-1}^{0\;STR}$, $F_{LTR}^{w}$, and $F_{STR}^{w}$, respectively. Figure 12 visualizes the normalized weights $s_{1}$ for selected channels of $F_{LTR}^{v}$, specifically using the 17th frame, locating in the middle of a GOP from Seq3. Due to the high dimensionality, we only display 7 channels at equal intervals for clarity. It can be observed that most channels effectively capture variations in the foreground, background, and objects with significant differences, thereby addressing the non-uniform characteristics across different spatial positions. The boundaries between the foreground, background, and objects with significant differences are typically regions of significant change and often attract greater human visual attention. The weight map’s ability to distinguish these regions facilitates visually consistent image reconstruction.

GOP size. To investigate the impact of GOP sizes, we evaluate the proposed LSVC-LTR under various GOP settings, including a scenario with only one initial intra-frame; i.e., the whole video is a GOP, denoted as GOP = −1 for brevity. Table 4 shows the comparison results. It can be observed that, as the GOP size increases up to 60, the proposed method demonstrates progressively larger improvements compared with EEM-4.1. However, the performance at GOP = −1 declines. This indicates that, while the proposed method performs well with larger GOP sizes, it does not function optimally when the GOP size is excessively large due to the attenuated temporal correlation. It can also observed that, for both traditional methods, such as HM-18.0 and VTM-22.0, and the LVC approach LVC-LTR, the BD-rate drops sharply as the GOP size increases. This is primarily due to the aforementioned error accumulation inherent in the LDP configuration. Similar to traditional approaches, periodically inserting an intra-frame can effectively alleviate such performance degradation.

Comparison with Two STRs. We also compare the performance of our proposed method with two STRs. Specifically, we modify EEM-4.1 to utilize the two preceding frames of the current frame as STR frames. The primary distinction between the two STR methods and our proposed LSVC-LTR lies in the substitution of the LTR frame with an additional STR. In addition, we remove the LSMA module and the historical guidance from both methods. To ensure a fair comparison, the training strategy remains the same. The test results are presented in Table 5. LSVC-LTR demonstrates performance improvements for Seq1-Seq4 and Seq6 while showing a loss for the remaining sequences, compared to the two-STR approach. This is attributed to the relatively low temporal redundancy in those specific sequences. These findings highlight the following advantages of using an LTR: (1) long-term reference frames generally exhibit higher image quality, providing a more stable and superior reference for reconstruction; (2) LTRs effectively mitigate the issue of error accumulation that typically arises with short-term reference frames.

Performance on General Dataset. To further explore the performance of the proposed LTR mechanism, we also evaluate the results on natural video datasets, i.e., HEVC test sequences, including Class B, Class C, Class D, and Class E. The results are shown in Table 6. Compared to EEM-4.1, the proposed method exhibits a 3.51% coding loss on Class B, while achieving bitrate savings of 2.28%, 5.80%, and 6.06% on Classes C, D, and E, respectively. These performance gains on partial natural sequences indicate that our method is also effective in scenarios with moderate motion intensity. The observation highlights a limitation of LSVC-LTR. This may be attributed to overfitting on the static data. Ideally, a well-designed and fully trained adapter should not, at the very least, degrade performance. In the future, we will further explore the underlying reasons, e.g., the adapter’s structure, its inherent mechanism compared with RDO, and the trade-offs between static and natural videos, to enhance generalization capability.

Statistical significance. To further validate the reliability of the proposed method, we conduct a paired *t*-test to compare the BD-rate savings of our approach and EEM-4.1 relative to the VTM-22.0 anchor across the test sequences. The statistical analysis demonstrates that our approach yields a significant improvement over EEM-4.1, with a mean BD-rate difference of −15.76% (t(7)=−2.901,p=0.0229). The 95% confidence interval for the mean difference is [−28.60%,−2.91%]. Furthermore, the effect size (Cohen’s d=−1.03) indicates a substantial practical impact. These results confirm that the performance gains of our method are consistent and statistically robust across different video content.

To address potential concerns regarding training variance in neural network-based models, we emphasize that the observed performance gain is not localized to specific sequences but is consistent across the entire test set. The calculated 95% confidence interval for the mean BD-rate difference between our method and EEM-4.1 does not cross zero, ranging from −28.60% to −2.91%. This provides strong evidence that the proposed architecture offers a reliable performance improvement rather being than a result of stochastic training artifacts.

## 6. Discussion

The proposed LSVC-LTR demonstrates that incorporating an additional long-term reference can significantly improve the overall compression efficiency of LVC for surveillance videos, even when only a single key frame is introduced. The improvements for the long–short motion adapter address the unequal importance between long-term and short-term motions. Furthermore, the historical motion vector guidance module confirms that utilizing available decoded motion information at the decoder can further improve efficiency. These findings distinguish our work from the approaches relying on only one reference, which restricts the exploitation of long–short correlations at the decoder. LSVC-LTR is essentially a multiple-reference approach characterized by highly unequal long and short motions, in contrast to general B-frame compression. Despite these advantages, LSVC-LTR requires 2.43× higher MACs compared to the baseline EEM-4.1 without LTR, which is less economical and requires further simplification. Additionally, the current LSVC-LTR results in performance loss on certain general datasets, indicating that further optimization is necessary, as LTR can also be applied to natural sequences and should ideally yield no loss under proper rate-distortion optimization.

## 7. Conclusions

This paper proposes an adaptive long–short temporal modeling for learned surveillance video coding utilizing long-term reference, addressing the unequal importance of the long and short reference frames. A baseline for learned surveillance video coding with long-term reference and long–short context mining is set up. Then a long–short motion adapter that employs soft attention weights is introduced to adaptively modulate long and short motions. Furthermore, a historical motion vector guidance module is employed to enhance the capability of the motion decoder. The experimental results demonstrate that the proposed approach achieves BD-rate performance of {−0.16%, −53.50%, −56.66%, and −13.89%} for {Y, U, V, and YUV} components over VVC in terms of PSNR. This significantly surpasses the {12.41%, −28.78%, −30.79%, and 1.86%} reported for the baseline EEM. Although it still lags behind the state-of-the-art, e.g., DCVC-FM, we believe that the proposed method is generalizable to other approaches using optical flow as a motion representation and can be integrated into them to achieve superior performance.

In the future, a key direction will involve a smart algorithm for long-term reference selection using frame-level attention mechanisms, or an adaptive LTR generation module to fuse historical frames into high-quality references. Such adaptive generation may also benefit natural videos, scene changes, and illuminative variations, thereby extending its application scenarios. Another direction could be the joint optical flow estimation between long-term and short-term references to achieve low complexity, while explicitly taking the motion bitrate into account during motion estimation.

## Figures and Tables

**Table 1:** The BD-rate (%) comparison with SOTA for YUV PSNR. VTM-22.0 is set as the anchor for all approaches.

Sequence	Component	HM-18.0	DCVC-DC [32]	DCVC-FM [33]	EEM-4.1	LSVC-LTR
Seq1	Y	32.01	−30.84	−29.39	19.49	2.81
	U	36.44	−48.54	−82.26	−40.22	−48.68
	V	50.00	−52.13	−79.25	−37.16	−44.54
	YUV	34.81	−35.71	−42.23	4.95	−9.54
Seq2	Y	25.27	−19.12	−20.38	20.18	4.62
	U	32.26	−40.63	−83.31	−27.56	−50.43
	V	46.74	−43.49	−82.35	−34.91	−58.40
	YUV	28.83	−24.85	−35.99	7.32	−10.14
Seq3	Y	26.06	−24.53	−26.22	29.76	7.87
	U	43.00	−41.86	−79.36	−24.89	−45.42
	V	56.19	−52.56	−82.24	−40.49	−56.91
	YUV	31.95	−30.20	−39.87	14.15	−6.89
Seq4	Y	21.86	−39.48	−39.90	11.08	−16.11
	U	10.91	−56.76	−85.75	54.84	−61.10
	V	16.88	−62.45	−87.07	57.70	−66.17
	YUV	19.87	−44.51	−51.53	22.38	−27.99
Seq5	Y	31.98	−20.94	−23.00	7.72	3.43
	U	85.88	−52.59	−87.53	−49.98	−55.92
	V	82.68	−56.46	−85.59	−49.25	−58.95
	YUV	45.05	−29.33	−38.89	−6.61	−11.79
Seq6	Y	26.31	−36.72	−35.52	1.11	−4.31
	U	24.00	−55.81	−82.75	−49.72	−60.56
	V	25.27	−55.18	−79.16	−44.48	−54.71
	YUV	25.89	−41.41	−46.88	−10.94	−17.64
Seq7	Y	23.52	−30.54	−35.07	4.71	−2.90
	U	24.85	−55.03	−87.81	−48.26	−58.06
	V	22.37	−56.16	−85.46	−47.95	−56.14
	YUV	23.54	−36.81	−47.96	−8.49	−16.45
Seq8	Y	39.94	−32.35	−36.88	5.26	3.31
	U	43.09	−40.69	−81.75	−44.42	−47.85
	V	107.49	−63.25	−87.33	−49.81	−57.48
	YUV	48.78	−37.25	−48.76	−7.83	−10.68
Average	Y	28.37	−29.31	−30.79	12.41	−0.16
	U	37.55	−48.99	−83.82	−28.78	−53.50
	V	50.95	−55.21	−83.56	−30.79	−56.66
	YUV	32.34	−35.01	−44.01	1.86	−13.89

**Table 2:** The BD-rate (%) comparison with SOTA for YUV MS-SSIM. VTM-22.0 is set as the anchor for all approaches.

Component	HM-18.0 [8]	DCVC-DC [32]	DCVC-FM [33]	EEM-4.1	LSVC-LTR (Ours)
Y	29.01	−35.12	−43.60	4.84	−11.15
U	27.91	−53.68	−68.96	3.33	−34.43
V	50.04	−58.84	−74.85	−3.21	−36.58
YUV	31.50	−40.40	−50.68	3.65	−17.24
MACs (G)	-	2642	2225	599	1457
Parameters (M)	-	18.44	17.02	11.08	13.81

**Table 3:** Ablation studies on the proposed modules. EEM-4.1 is set as the anchor for different settings.

Row Number	EEM-4.1	LTR with LSCM	Enhanced LTR	Historical MV Guidance	LSMA	BD-Rate (%)
1	*√*					0.00
2	*√*	*√*				−7.97
3	*√*	*√*	*√*			−13.84
4	*√*	*√*	*√*	*√*		−14.51
5	*√*	*√*	*√*	*√*	*√*	−16.54

**Table 4:** The BD-rate (%) comparison under different GOP size settings. EEM-4.1 is set as the anchor for all settings.

GOP Size	HM-18.0 [8]	VTM-22.0 [9]	LSVC-LTR (Ours)
GOP = 12	59.60	24.77	−6.15
GOP = 24	83.95	44.48	−8.79
GOP = 32	37.27	5.23	−9.31
GOP = 60	102.58	61.60	−11.22
GOP = −1	109.24	74.65	−7.04

**Table 5:** The BD-rate (%) comparison of the proposed method with the two-STR approach. EEM-4.1 is set as the anchor for the two settings.

Sequence	Two STRs	LTR
Seq1	−3.13	−3.51
Seq2	−10.31	−11.78
Seq3	−10.49	−15.92
Seq4	−5.88	−7.59
Seq5	0.26	4.73
Seq6	−2.84	−3.83
Seq7	−6.02	−4.93
Seq8	−4.48	−4.24
Average	−5.36	−5.88

**Table 6:** The BD-rate (%) performance on a general dataset, i.e., HEVC test sequences. EEM-4.1 is set as the anchor for different approaches.

Class	Class B	Class C	Class D	Class E
HM-18.0 [8]	56.55	52.08	56.35	27.65
VTM-22.0 [9]	1.12	11.44	18.53	−8.30
DCVC-DC [32]	−29.85	−37.08	−40.27	−38.46
DCVC-FM [33]	−36.20	−42.11	−44.03	−48.78
LSVC-LTR (Ours)	3.51	−2.28	−5.80	−6.06

## Data Availability

The datasets generated during and/or analyzed during the current study are available from the corresponding author on reasonable request.
